# Lifestyle decisions and climate mitigation: current action and behavioural intent of youth

**DOI:** 10.1007/s11027-021-09963-4

**Published:** 2021-07-26

**Authors:** Gary J. Pickering, Kaylee Schoen, Marta Botta

**Affiliations:** 1 Department of Biological Sciences, Brock University, 1812 Sir Isaac Brock Way, ON L2S 3A1 St. Catharines, Canada; 2 Department of Psychology, Brock University, 1812 Sir Isaac Brock Way, ON L2S 3A1 St. Catharines, Canada; 3 Environmental Sustainability Research Centre, Brock University, 1812 Sir Isaac Brock Way, ON L2S 3A1 St. Catharines, Canada; 4 Sustainability Research Centre, University of the Sunshine Coast, 90 Sippy Downs, QLD 4556 Australia

## Abstract

Youth carry the burden of a climate crisis not of their making, yet their accumulative lifestyle decisions will help determine the severity of future climate impacts. We surveyed 17–18 year old’s (*N* = 487) to establish their action stages for nine behaviours that vary in efficacy of greenhouse gas emission (GGE) reduction and the explanatory role of climate change (CC) knowledge, sociodemographic and belief factors. Acceptance of CC and its anthropogenic origins was high. However, the behaviours with the greatest potential for GGE savings (*have no children/one less child, no car or first/next car will be electric, eat less meat*) have the lowest uptake. Descriptive normative beliefs predicted intent to adopt all high-impact actions, while environmental locus of control, CC scepticism, knowledge of the relative efficacy of actions, religiosity and age were predictive of action stage for several mitigation behaviours (multinomial logistic regression). These findings inform policy and communication interventions that seek to mobilise youth in the global climate crisis response.

**Supplementary Information:**

The online version contains supplementary material available at 10.1007/s11027-021-09963-4.

## Introduction

### Context

While anthropogenic climate change (CC) is both widely acknowledged and generally understood, the global response to mitigation has been inadequate. For instance, recent estimates indicate that human activities have led to 1 °C of warming above pre-industrial levels (IPCC 2018), and at the current rate of greenhouse gas emissions (GGE), this will reach 1.5 °C between 2030 and 2052. The impact to both human and natural systems is expected to be catastrophic if warming continues to rise to 2 °C above pre-industrial levels, leaving a narrow window for the transformative societal changes needed to limit global warming to around 1.5 °C. While governments and businesses clearly must play their part (IPCC 2018; Pedersen and Lam 2018), meaningful policy change and the development and implementation of mitigating technology take time to enact. In contrast, individual behaviours can be adopted immediately (Dietz et al. 2009) and are necessary given the significant proportion of emissions in Western countries that are directly attributable to individual actions and lifestyle decisions (Pacala and Socolow 2004; Dietz et al., 2009; Baiocchi et al., 2010; IPCC 2018; van de Ven et al., 2018; Moran et al., 2020). Understanding the predictors of, and barriers to, effective change at the individual level is critical. This is particularly the case for youth, who are the product of a fundamental shift this century in thinking about CC, must carry the burden of a climate crisis not of their making, and whose accumulative choices and decisions over the next several decades will help determine the diversity and quality of life on our planet.

### Youth and mitigation behaviour

Engaging youth to take up and adapt to a low carbon lifestyle is an important component of limiting global temperature increase to below 1.5 °C above pre-industrial levels, and requires youth to adopt a lifestyle of around 2–2.5 tons of CO_2_ emission per year by 2030 and an even smaller 0.7 tons by 2050 (United Nations, 2020). Importantly, youth and particularly late adolescents are at a developmental stage where they are developing worldviews that will influence values, attitudes and lifestyles for a lifetime (Gidley et al., 2004). This is also a period where behavioural habits, including those relevant to environmental stewardship, are being formed (Palupi and Sawitri 2018).

Individual-level climate mitigation behaviours vary in their relative efficacy in reducing GGE (e.g., Gardner and Stern 2008; Girod et al., 2014; Lacroix 2018). Recently, Wynes and Nicholas (2017) compiled existing literature and presented calculations that allow for mitigation actions to be categorised as high, moderate or low impact, based on their potential for reducing GGE. Of the actions assessed in that report, seven were classified as high-impact (> 0.8 tonnes CO_2_-equivalent [tCO_2e_] emission reductions per year), specifically—in descending order of impact—*have one fewer child*, *live car free*, avoid one transatlantic flight, buy green energy, *buy more efficient car*, switch electric car to car free and *eat a plant-based diet*. Five actions were classified as moderate- impact (0.2—0.8 tCO_2e_): replace gasoline with hybrid car, wash clothes in cold water, *recycle* and hang dry clothes, and one as low-impact (< 0.2 tCO_2e_): upgrade light bulbs. Actions italicized above are considered in the current study, along with the following behaviours for which youth have reasonable agency over: taking public transportation, conserving energy in the home, conserving water, vacation locally and avoiding products with excessive packaging.

One of the advantages of classifying the efficacy of climate mitigation behaviours in this manner is that it can guide climate educators, policy makers and communicators in designing interventions for youth that will assist in maximising the impact of their actions. For instance, Canadian high schools tend to under-report or fail to mention high-impact behaviours (Wynes and Nicholas 2017), and focus on the scientific foundation of CC rather than personal mitigation options (Wynes and Nicholas 2018). Correspondingly, knowledge of the relative efficacy of the various climate mitigation behaviours is generally poor amongst 17–18-year old’s, and several recommendations have been made for curricula content developers (Pickering et al., 2020b). It remains to be determined if this knowledge gap in youth is reflected in their current or planned behaviour, and this is one of the aims of the current study. While accurate knowledge of the relative efficacy of mitigation actions is needed to assist youth in making informed lifestyle decisions that support the GGE reduction targets, education alone is unlikely to be sufficient. That is, in contrast with the information-deficit model (Bulkeley 2000), personal and public action is associated with a range of interconnected perceptual and social factors (Barr and Gilg 2007). The current study builds on this prior work by determining which climate mitigation actions youth are currently performing and planning, and how these action stages are associated with beliefs, values, knowledge and sociodemographic factors.

### Barriers to change

The theory of planned behaviour (Ajzen 1991) represents a useful model for understanding broadly the determinants of behavioural intent. Under the theory of planned behaviour, behavioural intention is determined by an individual’s control over an action, normative beliefs or social pressures, and behavioural beliefs or attitudes (Ajzen 1991, 2002). If an individual has control over a specific action, feels socially pressured to carry out the action and has a generally positive attitude towards it, they are more likely to possess the intent to carry out that behaviour. The theory of planned behaviour has been successfully applied previously to help explain pro-environmental actions (e.g., Tonglet et al., 2004), including those undertaken by youth (Leeuw et al., 2015). Changes in behaviour typically occur over time through a series of different stages (Prochaska and Velicer 1997), and under the Transtheoretical Model—originally applied to health behaviour—the first four stages are conceptualised as individuals moving from the precontemplation stage (no intention of taking action in the near future) through contemplation (intention to change), preparation (intention to take action in the immediate future) and then to action (behaviour and lifestyle have changed (Prochaska and Velicer 1997; Prochaska et al., 2008). Tobler et al., (2011) have applied this approach to understanding willingness to adopt ecological food consumption behaviour. However, as previously noted, even when there exists an intention to change, specific barriers—including habitual and perceptual—may prevent or delay transition into the action stage (Tobler et al., 2011). A fuller understanding of these barriers is needed in the context of youth and climate mitigation.

In their study of pro-environmental behaviour in high-school students, Leeuw et al., (2015) reported that it is particularly important that youth have perceived control over the action and that they see significant others (family, friends and celebrities) performing it, in general agreement with the environmental action of young Australians (Fielding and Head 2012). Additionally, the latter authors concluded that youth with a greater internal locus of control and higher environmental knowledge display stronger pro-environmental behaviour and intentions. Whether these factors also associate with climate mitigation action in youth remains to be determined and is a focus of the current study. Several belief and values factors present as barriers to climate mitigation in adult populations but have been underreported in youth. For instance, in adults a conservative political identity predicts behaviours that generate more GGE, including higher meat consumption (Dhont and Hodson 2014) and less use of energy efficient technology (Gromet et al., 2013). This may be linked to the strong correlation between CC scepticism and political conservatism, which as an identity marker strongly influences beliefs as well as the likelihood of seeking out new information (Pickering, 2015; Rutjens et al., 2018; Munoz-Carrier et al., 2020). CC scepticism and uncertainty have been shown in adult samples to promote environmental inaction, including limiting climate mitigation behaviour (e.g., Hine and Gifford 1996; Gifford 2011). If one does not believe a problem exists, there is little motivation to change behaviour to help address it. A potentially important nuance of CC scepticism is the degree of acceptance of the role of humans in its origin. For instance, a recent report has shown that participation in organic waste diversion programs—a CC mitigating individual-level activity—is higher in adults with greater recognition of the anthropogenic origins of CC and not dependent on environmental values (Pickering et al., 2020a). Religiosity has also been linked with CC attitudes and behaviour in adult populations (Tjernström and Tietenberg 2008; Smith and Leiserowitz 2013; Morrison et al., 2015). For instance, within the Judeo-Christian system—the dominant religious grouping in Canada (Lipka 2018)—believers with a more literal interpretation of the bible are less likely to believe that CC is occurring, that it is caused by human activities and that their individual actions can make a difference (Morrison et al., 2015).

Consideration of the extent to which these beliefs and knowledge factors influence mitigation behaviour and intent amongst youth is needed to inform how to best engage them and help equip them with the resources needed to meet the climate challenges.

### Current study

Our study is primarily exploratory in nature and seeks to use a description of youth knowledge, attitudes and beliefs around CC to better understand the context for their mitigation action/inaction and to inform past research in this area. As such, our primary objectives are to assess(i) current CC mitigation behaviour and intent, and (ii) the predictors of action stage for CC mitigation in youth. A secondary objective is to better understand levels of CC knowledge and scepticism. We have selected 17–18-year-old individuals as our cohort as these youth are in the process of forming their own beliefs, identities and habitual behaviours that impact climate and environmental sustainability more broadly. Additionally, most 17–18-year-olds are still in a school setting and thus can be influenced by the CC curricular. CC mitigation action in this study is defined as that which reduces GGE (Pickering et al., 2020b). We are particularly interested in beliefs and action stages around high-moderate impact behaviours, as calculated by Wynes and Nicholas (2017), as their adoption can be expected to make the largest individual-level contribution to meeting international GGE reduction targets (Wynes and Nicholas 2017; IPCC 2018), and the need for further research on the barriers that limit their uptake has been identified (Wynes and Nicholas 2018). Similarly, Nielsen et al., (2021) have recently called for more research on the predictors of high-impact behaviours. An understanding of the knowledge, belief and attitudinal barriers to adopting these actions is very limited in the context of youth and is an important research gap addressed in this study.

Here, we expand on Pickering et al., (2020b) by presenting data from that study for the first time on climate mitigation action and intent amongst youth. We hypothesize that current mitigation action is mainly limited to lower-impact behaviours (H_1_), consistent with the foci of high school science textbooks (Wynes and Nicholas 2017) and limited knowledge of the relative efficacy of mitigation actions (Pickering et al., 2020b). Loosely informed by the theory of planned behaviour, we further expect that descriptive normative beliefs (H_2_; Leeuw et al., 2015), a higher internal locus of control (H_3_; Fielding and Head 2012), and low level of climate change scepticism (H_4_; Gifford 2011) will predict action stage for high-impact behaviours. Identifying the barriers associated with behavioural inaction or limited action toward climate mitigation and the factors that facilitate effective behaviour is critical for informing educational, policy and communication interventions for youth as part of an integrated and inclusive response to the climate crisis.

## Materials and methods

### Recruitment

Individuals were recruited through the online data collection company Dynata®. To be eligible, participants had to be between 17 and 18 years of age and Canadian citizens or permanent residents. Respondents aged 17 also had to obtain parental permission prior to completing the survey. Participants completed a survey distributed through the Qualtrics® platform, and ethics clearance was granted through the Brock University Research Ethics Board (# 17–360). Some participants did not answer all the questions; completed responses retained for analysis purposes varied between 463 and 487, depending on the question.

### Knowledge and beliefs

Subjective and objective CC knowledge were assessed as detailed in Table 1. For the former, scores from the two indicator statements were averaged to form the measure of subjective CC knowledge. For objective CC knowledge, a score was created following the method of Liu et al., (2017) corresponding to the proportion of correct answers (0% represents no correct responses and 100% represents correct responses to all questions). CC scepticism/uncertainty and acceptance of its anthropogenic origins were assessed as summarised in Table 1. Values for CC scepticism/uncertainty were averaged across the four indicator statements to create a scepticism score, where higher scores indicate higher scepticism.

**Table 1:** Measures of knowledge, beliefs and psychological traits

Measure	Indicator statements	Scale type		Response options and coding	Cronbach’s alpha
Subjective climate change (CC) knowledge^1^	*I have confidence in my knowledge about climate change in general; I have confidence in my knowledge about which behaviours to change to best reduce greenhouse gas emissions*	5-point Likert		Strongly agree (5) to strongly disagree (1)	0.76
Objective CC knowledge^2^	*Nitrous Oxide is a greenhouse gas* (true); *The major cause of increased atmospheric concentration of greenhouse gases is human burning of fossil fuels* (true); *Biological diversity will increase as global temperature increases* (false); *Scientists agree that, as a result of global warming, the sea level will continue to rise for at least a century* (true); *Aerosols are airborne particles that are known to contribute to the formation of clouds and precipitation* (true); *There is scientific consensus that there will be an increase in global precipitation as a result of global climate change* (true)	Categorical		True (1), False (0), Don’t know (0)	0.43
Knowledge of efficacy of CC mitigating actions	An efficacy knowledge score (EKS) for each participant was extracted from Pickering et al., (2020b). Refer text for full details	Six actions that reduce GGE were ranked (1–6) for relative efficacy & compared with actual efficacy (Wynes & Nicholas, 2017)			-
Acceptance of CC	*As far as you know, do you personally believe the world’s climate is changing or not?*	Categorical	Yes, No, Don’t know		-
CC uncertainty and skepticism^3^	Four statements—see Online Appendices	5-point Likert	Strongly agree (5) to Strongly disagree (1)		0.63
Origins of CC	See Online Appendices	*Which best describes your opinion?*	Five statements varying in degree of anthropogenic v natural origins of CC		-
Locus of Control^4^	*My individual actions can make a difference to the environment; I can influence decisions now, that will help protect the environment in the future; I am only one person, I can’t make a difference to the environment*	5-point Likert	Strongly agree (5) to Strongly disagree (1)		0.55
Religious exclusivity^5^	Four questions—See Online Appendices	Mixed dichotomous	Yes/No (Q1-2); Select one (Q3); Agree/Disagree (Q4)		0.63
Religious salience^5^	Three questions—See Online Appendices	Mixed	Select from two options (Q1); Yes/No (Q2); Not at all important to Extremely important (Q3)		0.75^*^
Descriptive normative beliefs^6^	1. *Rate the likelihood that these individuals will live car-free or switch to an electric car in the next year* (My parents/primary caregivers; My favourite teacher; My favourite celebrity). 2. *Rate the likelihood that these individuals will adopt a plant-based diet in the next year* (One or more of my parents/primary caregivers; One or more of my immediate family members; One or more of my extended family members; One or more of my close friends; One or more of my favourite celebrities). 3. *Rate the likelihood that the following individuals will have one less child due to* ***environmental concerns*** (My parents/primary caregivers; My best friend; My favourite celebrity)	5-point Likert	Extremely unlikely to Extremely likely plus ‘already doing it’ option		-

1 Pickering et al. (2020b); 2 Stoutenborough and Vedlitz (2014); 3 Poortinga et al. (2011); 4 Fielding and Head (2012); 5 Pearce et al. (2017); 6 Leeuw et al. (2015); *Standardised Cronbach’s alpha (mixed scales)

An efficacy knowledge score (EKS) was also determined as follows. Pickering et al., (2020b) had participants rank the efficacy of greenhouse gas emission (GGE) reducing behaviours (*have one fewer child, live car free, switch from electric car to car free, eat plant based diet, recycle, upgrade lightbulbs*) on a scale from 1 (the most effective) to 6 (the least effective). They then determined an EKS for each participant by taking the absolute value of the difference between a respondent’s ranking of each action and the ‘true’ rank from Wynes and Nicholas (2017), and then calculating the mean of these differences for each respondent. Under their scheme, a score of zero represents perfect alignment with the ‘true’ ranks (high knowledge) and three represents the maximum discordance in ranks (low knowledge). In order to make these values more intuitive—i.e., a higher score corresponding to greater knowledge—we performed two transformations to those scores. First, we subtracted the highest possible score (3) from each participant’s EKS, and then we multiplied the sum by − 33.333. This resulted in a maximum score of 100 (perfect agreement with the actual ranks; i.e., high knowledge) and a minimum score of zero (perfect disagreement with actual efficacy ranks; i.e. low knowledge).

Locus of control (LOC) with respect to environmental actions was assessed as outlined in Table 1, with responses reverse coded as needed. Responses across the indicator statements were averaged to create a final score whereby a higher value indicates a greater internal LOC. Belief in individual agency was further assessed with respect to CC mitigation specifically with the question *Do you believe that your activities or lifestyle choices can help to lessen climate change*?, with a *yes* or *no* response option. Religious affiliation was determined by participants indicating what religion they practice from the following options: *Catholic; Christian-orthodox; Protestant; Christian- other; Buddhist; Hindu; Jewish; Muslim; Sikh; Eastern Religion; Other religion; No religious affiliation.* Respondents who indicated a religion were then prompted to answer questions about the importance of religion in their lives. Specifically, the dimensions of religious exclusivity (views that there are definite rights and wrongs) and religious salience (place in an individual’s hierarchy that religion holds) were assessed using the measures of Pearce et al., (2017), as outlined in Table 1. Average responses for each of the two dimensions were calculated, with higher values indicating greater religiosity.

### Mitigation behaviour

Participants were asked to indicate their ‘current stage for each of the following behaviours’: *Eat less meat; vacation locally instead of flying to destination, recycle, avoid products with excessive packaging, conserve energy in the home, conserve water, take public transportation, my first/next car will be electric instead of fossil fuel, have fewer children.* These behaviours were generally based on the Wynes and Nicholas (2017) study, which calculated the relative effectiveness of these actions in reducing GGE. Response options (and categorisation of behaviour into discrete stages) followed the approach of Tobler et al., (2011): *I am not doing this and I am not willing to* (Precontemplation); *I would like to do this, but I do not know how* (Contemplation); *I would like to do this, and I already know how to start* (Preparation); *I am doing this already* (Action). In addition to these response options, *I do not intend to purchase a car* was added for ‘my first/next car will be electric instead of fossil fuel’, and *I do not intend on having children* was added for ‘have less children’.

In order to assess the role of social norms in predicting respondents’ behavioural stage for high-impact CC mitigation actions, we determined descriptive normative beliefs using the approach of Leeuw et al., (2015) by asking youth to rate the likelihood that specific referent others would perform the behaviour within the next year due to environmental concerns (5-point Likert scale—extremely unlikely to extremely likely, plus ‘already doing it’ option). Actions and referent others were *live car-free or switch to an electric car* (parents/primary caregivers, favourite teacher, favourite celebrity), *adopt a plant-based diet* (one or more of immediate family members, one or more of extended family members, one or more of close friends, one or more of favourite celebrities), and *have one less child* (parents/primary caregivers, my best friend, favourite celebrity). For the latter behaviour, ‘already done having children for other reasons’ was included as a response option for parents/primary caregivers*.*

### Other measures

Simple demographic measures were determined, including age, gender, location and political affiliation (*Who would you vote for in an upcoming election?*). Political orientation was further examined with the question *How would you best describe your political beliefs?*: *Extremely liberal, Liberal, Slightly liberal, Moderate, Slightly conservative, Conservative, Extremely conservative, Do not know.* Finally, participants were asked what best describes their current diet: omnivore (regularly eat red meat, fish or chicken), flexitarian (consciously consume a limited quantity of either all types or specific types of meat), vegetarian (totally limit the consumption of meat and fish) or vegan (consume no animal products) (adapted from Cliceri et al., 2018).

### Data treatment and analysis—general approach

Data was treated and analysed using XLSTAT Premium Version 2017.1.1 (Addinsoft, NY, USA). Climate change knowledge and beliefs were summarised with descriptive statistics, and their interrelationship and variation with sociodemographic and other factors were assessed with correlation analysis, chi-square and t-tests as described more fully in Sect. 3.2. Summary statistics were used to describe the different stages of action for each climate mitigation behaviour. Multinomial logistic regression analysis was used to determine the role of sociodemographic, knowledge, belief and agency variables (Table 3) in predicting action stages for the nine climate mitigation behaviours assessed, following the approach of Tobler et al., (2011), and as described fully in Sect. 3.4.

## Results

### General characteristics

Four hundred seventy-seven responses were recorded, with a mean age of 17.6 years. Table 2 shows the demographic characteristics of the sample. The sample is similar to the wider Canadian population for gender and province, although Quebec is somewhat under-represented (23% of population) and Ontario is somewhat over-represented (38% of population; Statista 2016). Many of the youth surveyed indicated that they did not know who they would vote for in an upcoming election. Of decided voters, the relative proportions reported for each party are similar to those of the wider Canadian population, except for Green, which is over-represented (Elections Canada, 2019). Religious affiliation is very similar to the wider Canadian population, except for None which is over-represented (29% of population; Lipka 2018). Diet is fairly representative of meat consumption patterns for the Canadian population, although frequency of the flexitarian diet has not been widely reported (Statista 2016).

**Table 2:** Characteristics of sample

		Number	Proportion of sample
Age	18 yrs	291	61%
	17 yrs	186	39%
Gender	Female	258	54%
	Male	200	42%
	Other/did not identify	19	4%
Province/territory	Ontario	224	47%
	Quebec	76	16%
	Alberta	10	12%
	British Columbia	10	12%
	Manitoba	19	4%
	Nova Scotia	14	3%
	Saskatchewan	14	3%
	Newfoundland and Labrador	10	2%
	New Brunswick	10	2%
	Other	1	0.2%
Political affiliation	Do not know	129	32%
	Liberal Party of Canada	82	20%
	Conservative Party of Canada	80	20%
	New Democratic Party	42	10%
	Green	39	10%
	None & other	34	8%
Religion	None	169	37%
	Catholic	119	26%
	Other Christian and Protestant	105	23%
	Other	64	14%
Diet	Omnivore	301	63%
	Flexitarian	129	27%
	Vegetarian	33	7%
	Vegan	14	3%

### Knowledge, beliefs and agency

#### How do climate knowledge and agency relate?

As previously reported by Pickering et al., (2020b) for the same sample, 88% of respondents (*n* = 429) believed that their activities or lifestyle choices can help to lessen climate change, while 12% (*n* = 58) did not. The average proportion of correct responses for objective CC knowledge was 59.3% (SD = 24); refer to Online Appendices for the distribution. We expected that confidence in CC knowledge would associate with objective CC knowledge; a positive correlation was indeed found between objective and subjective knowledge, r(455) = 0.23, *p* < 0.001. We also expected that individuals who believe their activities or lifestyle choices can lessen climate change would have greater objective CC knowledge. A biserial correlation showed that objective CC knowledge score was positively associated with believe in the efficacy of individual actions/lifestyle choices, r(455) = 0.21, *p* < 0.001.

#### Does acceptance of CC and its anthropogenic origins associate with sense of agency?

The average CC scepticism score was 2.38 (SD = 0.79), consistent with the Canadian adult population (Pickering 2015). The distribution of scores is given in the Online Appendices. Confirming a generally low level of CC scepticism in our sample, 90% of participants responded *Yes* to the question “*Do You Personally Believe the World's Climate is Changing or Not?” (*5%, *No*; 4%, *Don’t Know*)*.* We expected that individuals who were less sceptical of CC would be more likely to believe that their activities or lifestyle choices can help lessen it. A *t* test confirmed this hypothesis, t(456) = 3.32, *p* < 0.001, with an average scepticism score of 2.33 (SE = 0.04) for youth who believe their activities/lifestyle choices can lessen CC compared with 2.71 (SE = 0.10) for those who do not.

With respect to belief around the origin of CC, responses to the four options were: *Entirely caused by natural processes*, 6% (*n* = 29); *Mainly caused by natural processes*, 9% (*n* = 42); *Partly caused by natural processes and partly caused by human activity*, 28% (*n* = 131); *Mainly caused by human activity*, 44% (*n* = 207); and *Completely caused by human activity*, 12% (*n* = 57). To examine the hypothesis that youth with stronger belief in the anthropogenic origins of CC are more likely to believe that their activities or lifestyle choices can help lessen it, we collapsed responses into three categories *(mainly or completely caused by human activity, n* = 263; *partly caused by natural processes and partly caused by human activity, n* = 131; *mainly or entirely caused by natural processes, n* = 71) and ran a chi-squared test. The result confirmed our expectations, χ^2^(*N* = 466) = 7.0, *p* < 0.05, with proportionally more youth who believe their activities/lifestyle choices can help lessen CC represented in the *mainly or completely caused by human activity* option than youth who do not believe their activities/lifestyle choices can help lessen CC (Fisher’s exact test).

#### Do political affiliation or religiosity matter?

We expected that youth with a conservative political identity would have higher CC scepticism. A *t* test confirmed this hypothesis, t(281) = 22.29, *p* < 0.0001, with an average scepticism score of 2.70 (SD = 0.91) for youth who intend to vote for the Conservative Party of Canada and 2.19 (SD = 0.81) for those who would vote for any other party, in agreement with the association between political conservatism and greater CC scepticism reported in adult populations (Whitmarsh et al., 2011; Leiserowitz et al., 2014; Pickering 2015).

Finally, we also expected that youth for whom religion was more important in their daily life (*How important or unimportant is religious faith in shaping how you live your daily life*?; not important at all [1] to extremely important [5]) would have greater CC scepticism. A Pearson’s correlation analysis confirmed a small, positive relationship between importance of religion in daily life and CC scepticism, r(285) = 0.17, *p* < 0.01, in agreement with Morrison et al., (2015). Similarly, religious exclusivity was positively correlated with CC scepticism, r(286) = 0.16, *p* < 0.01.

#### What level of control do youth feel they have over environmental issues?

Locus of control with respect to environmental issues (LOC) was assessed on a 5-point Likert scale, with a higher average score indicating a higher internal LOC and a lower score indicating a greater external LOC. Overall, participants had an average LOC of 3.9 (SD = 0.80), which was very similar to the value reported for Australian youth (Fielding and Head 2012) and suggesting a more internally-orientated LOC. The distribution of scores is shown in the Online Appendices. We expected that individuals who believe their individual actions can contribute to climate mitigation would have a greater internal locus of control. A *t* test confirmed this hypothesis, t(470) = 89.48, *p* < 0.0001, with an LOC score of 4.02 (SD = 0.71) for youth who believe their individual actions can contribute to climate mitigation, and a score of 3.04 (SD = 0.81) for those who do not.

### Current mitigation action stage

**Fig. 1:**
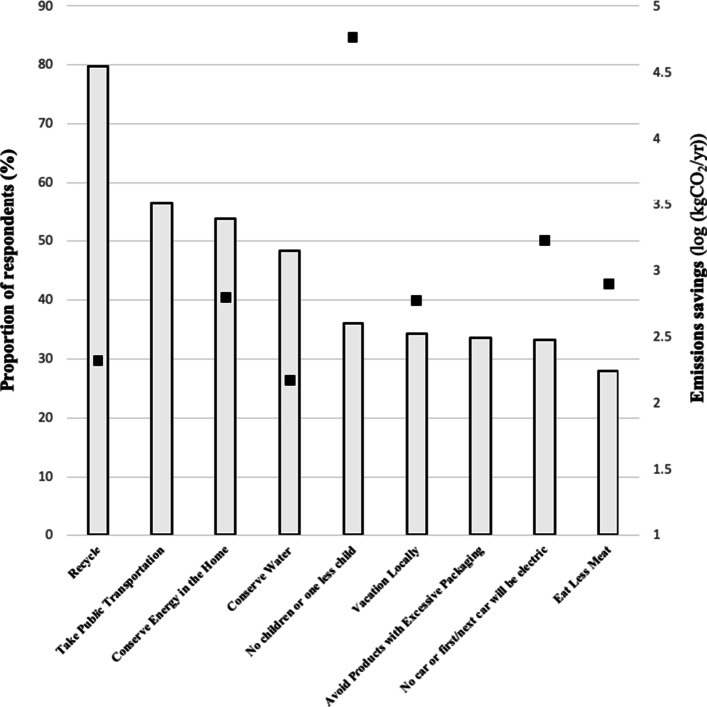
Mitigation behaviour of youth: proportion currently performing each action (*N* = 461–463) and potential savings in greenhouse gas emissions (square symbols). Note that savings are the mean values from Wynes and Nicholas (2017) expressed as logged values and increase by a factor of 10 with each log unit

Current stage of action for specific climate mitigation behaviours was assessed and categorised as *Precontemplation* (‘I am not doing this and I am not willing to’), *Contemplation* (‘I would like to do this, but I do not know how’), *Preparation* (‘I would like to do this, and I already know how to start’) and *Action* (‘I am doing this already’). Figure 1 shows the proportion of youth who indicate they are currently in the *Action* stage for specific CC mitigation behaviours. For illustrative purposes, we also include on the secondary y-axis estimates of the potential savings per year in CO_2_ emissions for each behaviour. Most estimates are based on Wynes and Nicholas (2017); refer to the Online Appendices for details of the calculations used. The average number of these behaviours that respondents reported that they are currently performing was 4.0 (SD = 2.0), and these counts approximated a normal distribution (Online Appendices). Only three actions are reported by the majority of respondents (*recycling*, 79.8%; *taking public transport*, 56.5%; *conserving energy in the home*, 53.8%), while *eating less meat* has the lowest proportion (28.0%). Noteworthy, the behaviours with the highest potential GGE savings have the lowest uptake by youth.

**Fig. 2:**
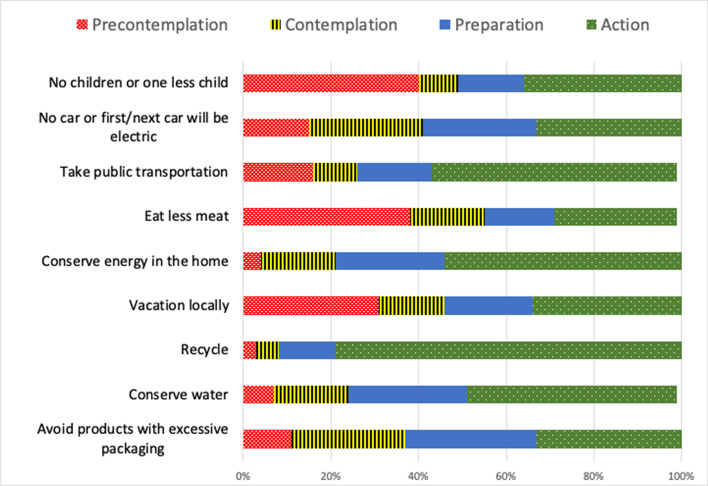
Action stage of youth for climate mitigation behaviours. Data shows proportion of respondents in each action stage (*N* = 461)

Figure 2 shows the responses for all four action stages. Unwillingness to perform in the future (precontemplation) was highest for *have no children/one less child* (40%) and *eat less meat* (38%). For the change stage (contemplation and preparation), *Avoid products with excessive packaging* (56%) and *No car or first/next car will be electric* (52%) had the highest proportion. This is a result which also held for the contemplation stage for both these actions (26%), indicating that respondents saw the greatest need for more information/education around these behaviours in order to operationalise their intent. Change was lowest for *Recycle* (18%), likely reflecting the high proportion of individuals already doing this.

### Predictors of mitigation behaviour

Here, we sought to better understand how knowledge, beliefs and sociodemographic factors associate with action stages. We were particularly interested in higher-impact behaviours identified by Wynes and Nicholas (2017), including *having one fewer child, switch to an electric car or live car free* and *eat a plant-based diet/eat less meat*, which can realise GGE savings (tCO_2_/yr) of up to 58.6, 1.7 and 0.8, respectively (refer Online Appendices). Our analyses followed the approach of Tobler et al., (2011), whereby multinomial logistic regressions were run for each behaviour comparing youth in the precontemplation and action stages with those in the change stages (contemplation or preparation) as the reference category. For high-impact behaviours, we also considered the role of descriptive normative beliefs (‘social norms’); the likelihood that specific referent others would perform the action within the next year due to environmental concerns or were already performing it. A social norm score was calculated separately for each high-impact behaviour by averaging the responses from the 5-point Likert scale for each referent other (extremely unlikely (1) to extremely likely or ‘already doing it’ (5); Leeuw et al., 2015). Refer to the Online Appendices for a summary of these data. Table 3 provides a summary of the predictors for precontemplation and action stages, with full model parameters provided in the Online Appendices.

**Table 3:** Summary of findings from multinomial regressions for predicting action stage for climate mitigation behaviours. + and – symbols indicate the variable had a positive or negative effect, respectively, on membership of the specific action stage group (standardised β). (Refer Online Appendices for full model parameters)

		Sociodemographics			Climate knowledge			Beliefs and agency		
Mitigation behaviour	Action stage	*Gender (male)*	*Age*	*Religion (yes)*	*Subjective CC knowledge*	*Objective CC knowledge*	*EKS*	*Social norm*	*Locus of control*	*CC scepticism*
One fewer child	Precontemplation	ns	+	ns	ns	ns	ns	-	ns	ns
	Action	ns	ns	ns	ns	ns	ns	ns	ns	ns
First/next car electric	Precontemplation	ns	ns	ns	ns	ns	ns	-	ns	+
	Action	ns	ns	ns	ns	ns	+	-	ns	ns
Eat less red meat	Precontemplation	ns	+	ns	ns	ns	ns	-	ns	ns
	Action	-	ns	ns	+	ns	+	ns	+	ns
Recycle	Precontemplation	ns	ns	ns	ns	ns	ns	NA	ns	ns
	Action	ns	-	ns	ns	ns	ns	NA	ns	-
Take public transport	Precontemplation	ns	ns	ns	ns	ns	ns	NA	-	ns
	Action	ns	ns	ns	ns	ns	ns	NA	ns	-
Conserve energy in home	Precontemplation	ns	ns	ns	ns	ns	ns	NA	ns	+
	Action	ns	-	ns	ns	ns	ns	NA	ns	-
Conserve water	Precontemplation	ns	ns	ns	ns	ns	ns	NA	ns	ns
	Action	ns	-	ns	ns	ns	ns	NA	ns	ns
Vacation locally	Precontemplation	ns	+	ns	ns	ns	ns	NA	ns	ns
	Action	ns	ns	-	ns	ns	ns	NA	ns	ns
Avoid excessive packaging	Precontemplation	ns	ns	ns	ns	ns	ns	NA	-	ns
	Action	ns	-	ns	ns	+	ns	NA	+	ns

Reference category for multinomial regressions was the change (contemplation/preparation) stages; CC, climate change; EKS, Efficacy knowledge score (knowledge of the relative efficacy of mitigation actions); ns, not significant; NA, not assessed

#### Sociodemographics

Gender was predictive of just one of the nine behaviours, such that males were less likely than females to be in the action stage for *eat less red meat.* In contrast, age (17 or 18 years old) was predictive for two of the three high-impact behaviours and six of the seven lower-impact behaviours. The direction of the age effect was the same for all behaviours where it was significant; older respondents were more likely to be in the precontemplation stage, and younger respondents were more likely to be in the action stage. Identification with a religious affiliation was predictive of just one behaviour; individuals who did not identify with a religious affiliation were more likely to be in the action stage for *vacation locally*.

#### Climate knowledge

Subjective climate change knowledge associated with the high-impact behaviour of *eat less red meat*, such that higher subjective knowledge predicted greater likelihood of being in the action stage. Objective climate change knowledge only predicted *avoid excessive packaging*, whereby participants with higher knowledge scores were more likely to be in the action stage. Two of the three high-impact behaviours were predicted by knowledge of the relative efficacy of mitigation actions (EKS). Specifically, youth with higher EKSs were more likely to be in the action stages for both *first/next car will be electric* and *eat less red meat.*

#### Beliefs and agency

While social norm was only assessed for each of the three high-impact behaviours, importantly, it predicted all three. Specifically, individuals with lower descriptive normative beliefs were more likely to be in the precontemplation stage. In the case of *first/next car will be electric*, individuals with lower descriptive normative beliefs were also more likely to be in the action stage compared with the change stage, although this result only just reached significance (p(χ^2^) = 0.044). A higher internal locus of control for environmental actions predicted action stage for *eat less red meat*, *take public transport* and *avoid excessive packaging*. In all cases, youth with a higher LOC were more likely to be in the action stage, and/or youth with a lower LOC were more likely to be in the precontemplation stage. CC scepticism predicted action stage for *first/next car will be electric*, *recycle*, *take public transport* and *conserve energy in the home*. In all cases, participants who were more sceptical were more likely to be in the precontemplation stage and/or less likely to be in the action stage.

#### Religiosity

Respondents who identified with a religious affiliation (*n* = 282) also completed the measures of religious exclusivity and religious salience (Table 1). For these respondents, we ran separate multinomial logistic regressions with these two measures as the predictor variables, precontemplation and inaction as the dependent variables and change stages as the reference category. Neither religious exclusivity nor religious salience were significant for *have one fewer child, my first/next car will be electric, recycle, take public transport, vacation locally* or *avoid excessive packaging* (p(χ^2^) > 0.05). For *eat less meat* (action *n* = 76, change *n* = 106, precontemplation *n* = 100), religious exclusivity was significant (standardized β, -0.24; SE, 0.11; 95% CI, -0.44- -0.03; p(χ^2^) = 0.03), such that individuals with higher exclusivity scores were less likely to be in the action stage (OR, 0.29). For *conserve water* (action *n* = 138, change *n* = 123, precontemplation *n* = 21), religious salience was significant (standardized β, 0.35; SE, 0.09; 95% CI, 0.17-0.53; p(χ^2^) < 0.001), such that individuals with higher salience scores were more likely to be in the action stage (OR, 1.46). Finally, for *conserve energy in the home* (action *n* = 167, change *n* = 107, precontemplation *n* = 11), both religious salience (standardized β, 0.24; SE, 0.09; 95% CI, 0.07- 0.42; p(χ^2^) = 0.007), and exclusivity (standardized β, -0.18; SE, 0.09; 95% CI, -0.35- -0.01; p(χ^2^) = 0.04) were significant, such that individuals with higher exclusivity scores were less likely to be in the action stage (OR, 0.39) and those with higher salience scores were more likely (OR, 1.29).

## Discussion and conclusions

In Sect. 3.2 we considered our results on climate knowledge and beliefs within the context of current literature. In this section, we focus the discussion on the main study hypotheses (Sect. 1.4) and how our findings add to both existing literature and inform policy and educational planning.

### Current mitigation behaviour

The finding that youth are mainly engaging in lower-impact mitigation behaviours confirms H_1_ and is in accord with Pickering et al., (2020b), who reported for the same sample that youth generally have poor knowledge of the relative efficacy of GGE-reducing actions. The high uptake of the albeit lower efficacy behaviours—especially recycling—is encouraging at face value, signalling that youth are engaging in individual-level mitigation activities. However, the difference in emissions savings between these and higher efficacy behaviours is non-trivial with, for instance, the decision to have no children or one less child approximately 300 times more impactful than recycling (Murtaugh and Schlax 2009; Wynes and Nicholas 2017). The low prevalence of eating less meat might reflect relatively poor understanding in this cohort of the link between intensive meat farming and CO_2_ emissions, consistent with the limited knowledge of Canadian adults on the negative environmental impacts of industrialised red meat production (Stea and Pickering 2018). Further supporting this conjecture, in a recent survey of individual actions recommended for mitigating CC in Canadian high school science textbooks, only two recommended eating less meat, and none advocated eating a plant-based diet (Wynes and Nicholas 2017).

Females were more likely than males to be currently performing two of the three high-impact actions (*no car or first/next car will be electric* and *eat less meat*). This finding cannot be explained by CC scepticism, t(455) = 1.84, *p* = 0.18, recognition of the anthropogenic origins of CC, Χ^2^(2, *N* = 457) = 4.00, *p* = 0.13, CC knowledge, t(455) = 2.59, *p* = 0.11, nor knowledge of the efficacy of different mitigation actions (EKS; t(439) = 0.01, *p* = 0.93; Pickering et al., 2020b), as these did not vary with gender. Thus, considerations unrelated to CC perception or knowledge are likely underlying these gender differences in current mitigation behaviours. For instance, adolescent females eating less meat than males may be due to the greater emphasis they place on health, body image and weight concerns (Cooper and Goodyer 1997; Wardle et al., 2004).

Findings for the most impactful action—having no children or one less child—must be interpreted with some caution with respect to current behaviour, as 17–18 year old youth are not typically procreating at this age in most Western countries. Interestingly, however, one variable did predict greater likelihood of youth performing this action—identifying as a non-omnivore (flexitarian, vegetarian or vegan). Given that environmental concerns are one of the motivators for vegetarians adopting this dietary behaviour (Fox and Ward 2008), it is possible that these individuals are more likely to seek out or be more attentive to information related to other individual-level pro-environmental behaviours (in this case the climate implications associated with family planning).

Although they are limited to lower-impact behaviours, it is interesting that 17-year-olds are statistically more likely than 18-year olds to currently be performing four of the mitigation actions. The same trend was observed for the other five actions examined (data not shown), suggesting that this is a broadly applicable finding. This result cannot be explained by CC scepticism, t(457) = 0.06, *p* = 0.81, recognition of the anthropogenic origins of CC, Χ^2^ (2, *N* = 457) = 0.39, *p* = 0.82, CC knowledge, t(457) = 3.14, p = 0.08, nor belief that their activities or lifestyle choices can help to lessen climate change, Χ^2^(1, *N* = 456) = 2.2, *p* = 0.14, as these did not vary with age. Interesting, however, as first reported by Pickering et al., (2020b) in this sample, 18-year olds had higher knowledge (EKS = 36.6, SE = 1.1) than 17-year olds (EKS = 31.0, SE = 1.4) on the relative efficacy of different mitigation actions, t(448) = 9.55, *p* < 0.01. This is often a period of significant transition and adjustment for 18-year-old youth in North America as they enter the full-time workplace for the first time or adapt to their first year at college. These changes often correspond with moving away from the family home and/or exposure to a range of new socioeconomic, psychological and operational stressors (reviewed in Bland et al., 2012), which we speculate may disrupt previous habitual mitigation behaviours, and may also alter socially normative influences around these activities.

### Predicting mitigation action stage

The three high-impact individual-level mitigation behaviours of *no children/one less child, first/next car will be electric* and *eat less meat* (Wynes and Nicholas 2017) were all predicted by descriptive normative beliefs, confirming H_2_. This finding agrees with the report of Leeuw et al., (2015), who found with a younger cohort (median age 13.6 yrs.) that descriptive norms had a moderate positive effect on intentions toward eco-friendly behaviours, whereas injunctive norms did not. That is, youth may be more influenced by what they believe significant others are doing or plan to do with respect to pro-environmental action, rather than what they believe they ought to be doing.

A higher internal locus of control (LOC) predicted action stage for one of the three high-impact behaviours examined (*eat less meat*), thus providing limited confirmation of H_3_. As informed by the study of wider pro-environmental behaviours and intentions of Fielding and Head (2012), we assessed LOC in part to reflect the sense of powerlessness that many youth experience in response to environmental challenges. The absence of a LOC effect for *no children/one less child* may be related to a lack of awareness around the environmental consequences of the action, as suggested by Pickering et al., (2020b). If youth do not understand that this behaviour links strongly with environmental and climate benefits, then perceived agency over pro-environmental outcomes would not be expected to predict intention to perform it. It is unclear, however, whether this explanation also helps to account for the null result for *no car or first/next car will be electric*. While reducing the effects of driving, such as buying a more fuel-efficient car, is recommended in the Government of Canada documents on how to reduce individual-level GGE, living car free is not (Wynes and Nicholas, 2017—Table 1). Similarly, Pickering et al., (2020b) reported for this sample that when 17–18 year olds were asked what the most effective actions they can take to lessen CC were, 24% indicted driving less, while only 6% cited using electric or hybrid cars. Our finding that belief that individual actions can contribute to climate mitigation correlates well with LOC scores related to the environment more broadly suggests that the latter measure may be useful in future quantitative research that seeks to examine CC mitigation and the role of individual agency. This could include a more in-depth examination of the related concept of self-efficacy—whether an individual believes that they possess the ability and skills to execute specific actions (Bandura 1977).

CC scepticism predicted action stage for one of the three high-impact actions examined (*no car or first/next car will be electric*), thus providing limited confirmation of H_4_. North America has been traditionally described as a car culture (Sparrow 2019), and as reported by Green et al., (2018), driving amongst youth is viewed as constitutive of adulthood, ‘… rites of passage on a normative path from childhood “dependence”’ (p18). Notably, the authors also comment that environmental sustainability was absent from youth discussions around car driving and ownership. It is possible that the CC scepticism finding in our study reflects a response to cognitive dissonance (Festinger 1957). That is, when the conflicting values/desires of care for the environment and car ownership aspirations are held, the conflict is resolved by invoking the belief that CC—and thus any contribution that cars might make—is uncertain or even non-existent. While CC scepticism trended in the hypothesized direction for all high-impact behaviours, it was low and showed limited variability in our sample, which may have contributed to the null result for the other behaviours examined.

Knowledge of the relative efficacy of individual-level mitigation behaviours predicted being in the action stage for *no car or first/next car will be electric* and *eat less meat*, although interestingly, objective CC knowledge did not (nor did it for five of the six lower-impact behaviours). Previous recent work has also used knowledge of behaviour efficacy when investigating CC behaviour and beliefs (e.g. Wynes et al., 2020). Our EKS and the related construct of carbon numeracy have the advantage of more directly linking action stage with arguably the most salient component of climate knowledge that related to mitigation behaviour. This is consistent with the findings of Shi et al., (2016) who reported that different types of climate knowledge have different effects on CC concern.

As alluded to in Sect. 4.1, age matters; it was predictive of the action stage for more behaviours—both high and low impact—than any other factor examined, despite the narrow age range of our cohort (17- and 18-year-olds). Additionally, the direction of this finding was the same for all behaviours where it was significant; younger youth were more likely to be in the action stage and older youth were more likely to be in the precontemplation stage. We speculated earlier that this may be related to the disruption in habitual mitigation behaviours or altered normative influences for 18-year-olds as they transition away from living at home. However, it may equally be due to cognitive and/or social factors that are more developmental in nature. Thus, we encourage further research to more fully elucidate this finding, as interventions aimed at sustaining or encouraging new pro-climate behaviours may need to differ depending on the age of the youth, even within the narrow band identified here.

Affiliation with a religion was not a major predictor of mitigation action states for youth, which may partly reflect the previously reported decline of both the importance of religion and formal religious affiliation amongst youth in North America (Pew Research Center 2018; Cox 2019). However, within the subset of youth who did report a religious affiliation in our study, religiosity (religious exclusivity and/or religious salience) was important for three behaviours, whereby higher exclusivity meant lower likelihood of being in the action stage and higher salience mean greater likelihood of being in the action stage. While concern about CC has been linked with religiosity in several studies, including across the 40 nations examined in Mostafa (2016), there is very limited literature on how religiosity translates to individual-level climate mitigation behaviour and no studies that we could identify on youth. While belief in man’s dominion over nature and the importance of environmental stewardship can vary between and within religions (Morrison et al., 2015), it has been reported that adults with a more literalist interpretation of scripture have lower concern about the environment (Guth et al., 1995; Gehlbach & Artino, 2018), and within the context of Christianity—the dominant religion in Canada (Pew Research Center 2018)—literalists are the least engaged and least likely to support CC action (Morrison et al., 2015). To the extent that participants with high religious exclusivity have more of a literalist bent, our data suggest that these findings may extend to both individual-level climate action and to youth, yet also indicate that the different dimensions of religiosity do not predict climate action in the same way.

### Policy implications and other application of findings

While some commentators have argued that wider uptake of lower-impact GGE-reducing actions might be the best strategy for maximising climate mitigation at the individual level (Stern and Wolske 2017), we contend that the climate crisis has escalated to a level where every effort should be made to facilitate adoption and maintenance of high-impact lifestyles to augment the similar scale of change needed from governments and industry. Youth are uniquely positioned to contribute to this challenge; indeed, they must. Several decisions and considerations related to the most effective behaviours for reducing GGE are being made/formed during late adolescence, including habitual dietary choices (CDC (Centers for Disease control and Prevention), 1996), the decision to acquire a driver’s licence and purchase a first car, becoming sexually active and considering the impact of childbearing. Additionally, the impact of not adopting a low carbon lifestyle will, on average, accumulate across a longer period for youth than for older individuals, leading to greater total net emissions. Adopting and sustaining the most impactful behaviours will be difficult and require on-going commitment (Stern and Wolske 2017), likely compounded by the knowledge that the climate crisis is not of their making and that they are being asked to make lifestyle sacrifices that previous generations were not able or willing to (Wynes and Nicholas 2018). Below we discuss how interventions around education, infrastructure and communications may be mobilised to assist with these challenges.

#### Education and agency

Our findings support enhancing CC education on the relative efficacy of individual-level GGE-reducing activities. This is especially salient for influencing action around high-impact behaviours, where EKS was predictive for two of the three activities. As noted by Wynes and Nicholas (2019), current secondary science learning objectives tend to place little emphasis on CC impacts and solutions, instead focusing on other topics such as physical climate mechanisms. Enhancements should focus on secondary school curricula introducing and contextualising the high-impact actions, despite anticipated resistance and political pressure applied from some religious (*no children or one less child*), fossil-fuel industry (*no car or first/next car will be electric*) and animal agriculture (*eat less meat*) special interest groups. Youth may simply not be aware of the extent to which these lifestyle choices affect GGE (Pickering et al., 2020b). Part of this contextualisation might include greater emphasis on numeracy skills, as suggested by Pickering et al., (2020b), whereby differences in the efficacy of GGE-reducing behaviours can be better understood, and youth can be less susceptible to framing manipulations and misinformation (Peters et al., 2006) within the politicised environment of the CC ‘debate’. These changes, however, should not come at the expense of ongoing education on the reality and certainty of CC, as CC scepticism/uncertainty was associated with action stages for several behaviours in our study.

Education can be effective by increasing agency amongst youth. When high school students both understand the causes of CC and have knowledge of specific action strategies, their sense of agency is increased, and they are more likely to engage in climate mitigation (McNeill and Vaughn 2010). We encourage approaches that reinforce the anthropogenic origin of CC, given that this associated in our study with belief that one’s individual activities/lifestyles can help lessen CC. Educational initiatives that stress the positive and significant effects that individual behaviours can have on the environment should also be encouraged, given that LOC was a significant predictor of the action stage for three mitigation behaviours, including currently eating less meat. Indeed, this latter action could form an appropriate case study as an exemplar for a more integrated, interdisciplinary environmental science curricula. The environmental impacts of intensive animal agriculture—which currently are poorly known amongst the general public (Stea and Pickering 2018)—might be incorporated in a module that then focuses on the corresponding GGEs and how they are calculated and culminate in a behavioural science component that considers demand behaviour economics, individual empowerment through personal decision-making and social and other obstacles affecting adoption of a reduced-meat or meat-free diet. If these and other educational approaches can increase youths' sense of agency toward CC, this may also decrease the fear and anxiety experienced in thinking about their future (Tonn and MacGregor 2009) and CC in particular (Clayton 2020). Climate anxiety is particularly prevalent among younger adults and teenagers (reviewed in Clayton 2020) and may lead to ‘eco-paralysis’, preventing individuals from engaging in climate action (Albrecht 2011).

Finally, these suggestions regarding curricular goals emphasize the importance of adopting an interdisciplinary approach to CC education. One additional benefit of adjusting curricula and its delivery, such as textbook content, to address the identified deficits is the potential influence on families. For instance, most 17–18-year-old Canadians (the cohort in this study) are still living with their parents, affording an opportunity for these youth to affect parental attitudes and action on mitigation—a reversal of the direction in which descriptive norms and modelling desirable behaviour are traditionally understood as operating.

#### Infrastructure, social norms and communications

While the majority of respondents are open to owning an electric rather than fossil-fuelled car—one of the high-impact mitigation behaviours (Wynes and Nicholas 2017)—many indicate that they do not know how, suggesting that accessibility is an issue for youth. Charging station coverage and related infrastructure are still limited in Canada, although recent government (Government of Canada 2020) and industry (Sarabia 2020) programs have dedicated resources to improving this. Our findings should encourage the expedition of these initiatives, given the high interest amongst youth in electric cars. However, the cost of these vehicles has been identified as the single greatest barrier for North Americans (Halvorson 2020), which may represent an even greater challenge for youth, given their relatively limited financial resources. Fiscal incentives such as subsidies, rebates or tax breaks are tools that governments might consider using to help make the purchase or leasing of electric vehicles more affordable for young people, as these approaches have proved effective in other areas of pro-environmental behaviour (Maki et al., 2016). Additionally, car sharing programs such as Zipcar offer a cheaper and popular alternative to purchasing for youth, and companies offering these services may be encouraged to ensure adequate stock of electric vehicles in their fleets.

Communication strategies aimed at encouraging adoption of meaningful mitigation behaviours may be most effective if they can exploit the importance to youth of descriptive normative beliefs. As reported by Elgaaied-Gambier et al., (2018) in an adult cohort, triggering a positive descriptive norm can lead to the adoption of pro-environmental behaviour (e.g., avoiding overpackaging), even when this norm does not reflect the behaviour of the majority, provided that consumers perceive the endorsement as credible. For norm-based communications to be effective with youth, they should focus on the behaviour of their significant others, which may include encouraging parents—as important youth role models (Martin and Bush 2000; Leeuw et al., 2015)—to set good examples. However, celebrity endorsement can be very influential among adolescents (Martin and Bush 2000; Chan et al., 2013), and innovative communication strategies that incorporate celebrities have the potential to elicit the desired behaviours. To be effective, however, celebrity selection needs to consider individuals who are clearly pro-environmental to ensure endorser-brand congruence (Chan et al., 2013; Blasche and Ketelaar 2015), and content should focus on descriptive rather than injunctive norms (Melnyk et al., 2010; Leeuw et al., 2015).

Celebrities who have adopted the three high-impact lifestyle behaviours—or a subset thereof—could be recruited and promoted, profiled and otherwise used to endorse the behaviour to youth through media campaigns, as has been used successfully for decades in the endorsement of big brand consumer products (Kamins and Gupta 1994). This approach has a precedent in the literature with young adults, with Inoue and Kent (2012) reporting higher pro-environmental intent after promotion by sports teams, although the behaviours tested were limited to recycling-related activities. It of course remains to be determined whether this is an optimum use of the typically limited budgets of government departments and environmental NGOs charged with facilitating climate mitigation, but we suggest it is worth exploring further. Such campaigns should target the media platforms that youth commonly use to consume their news and entertainment, particularly social media and the Internet (Pickering et al., 2020b). Additionally, incorporating celebrity lifestyle profiles and interviews as endorsements of the desired behaviours might also be effective as a component of a more integrated environmental science curriculum, as identified above.

High-impact behaviours confer their climate mitigation effects over time, and benefits are not often immediately noticeable. Youth do not readily receive social media validation for choosing to have fewer children, whereas they can post about re-useable straws and see immediate social benefit and encouragement (Stafford and Jones 2019). It can be argued that such validation provides necessary support in sustaining these actions and perhaps leads to spill-over effects whereby further behavioural changes are forthcoming. However, there is a concern that it might also serve simply to reinforce guilt alleviation (Parizeau et al., 2015; Schanes et al., 2018) and tokenism (Gifford 2011) and actually inhibit adoption of the necessary high-impact behaviours. Thus, we encourage the development of communication platforms and fora where youth can not only discuss the challenges and successes of meaningful lifestyle changes, but receive the social validation, support and other tools needed to sustain them. Concurrent with this should be initiatives that seek to provide social media influencers (Khalid et al., 2018) with the requisite knowledge of the efficacy of different GGE-reducing actions and the behavioural and social supports needed by their youth audiences.

### Limitations and other considerations

We did not measure environmental concern in our study, nor did we directly assess the motivators behind both current and intended action for the mitigation behaviours presented. For instance, to what extent is taking public transport a conscious effort to reduce GGE, or a simple necessity due to no other transport option being available? Additionally, an individual’s attitude toward an action is a component of predicting behavioural intent according to the theory of planned behaviour (Ajzen 1991, 2002). These considerations should be further elucidated, as they speak to a fuller understanding of the role of attitudes, including climate concern, in influencing specific mitigation actions and thus the optimal interventions to promote them in young people. This may be especially salient in the sensitive (Pedersen and Lam 2018), but important, case of family planning. Of the individual-level actions quantified to date, choosing to have no children or one less child represents the greatest contribution that can be made to reducing GGE (Wynes and Nicholas 2017). Yet, as noted earlier, this was the behaviour youth showed the highest resistance to. Qualitative methods, including individual interviews and focus groups (Caillaud and Kalampalikis 2013), may be particular helpful in eliciting the attitudes of youth around this lifestyle option, including assessment of perceived fairness, equity and human rights (Pedersen and Lam 2018; Pickering et al., 2020b). Finally, to tie future research more tightly into the theory of planned behaviour, level of control should be considered for each action. Here, as this was an exploratory study, we used one general locus of control measure for environmental action following the approach of Fielding and Head (2012).

The mitigation behaviours that youth responded to in our study were not intended to be exhaustive but were selected primarily on the basis of whether or not robust data was available in the literature on their relative efficacy. It is certainly possible that there are actions that youth have adopted, or are planning to, that they believe are mitigating but were not included. However, an examination of the free-text responses of youth to the question *In your opinion, what are the most effective actions that you could take to help lessen climate change in your life?* reported in Pickering et al., 2020b (Table 2) for the same sample indicates that we have captured the most salient behaviours. We did not measure descriptive normative beliefs for the low-moderate impact behaviours in our study; if this had been done, a more comprehensive consideration of the importance of this factor and how it may vary with action efficacy would have been possible. Finally, our choice of measures and response types in this study were largely informed by their prior use in the literature and by keeping the survey length manageable to increase completion rates and data quality. However, there are other approaches which may reduce measurement error, and the interested reader is referred to Gehlbach and Artino (2018) for a considered overview of survey design practises.

Conceptualising the action and intent of youth using the Transtheoretical Model (Prochaska et al., 2008) as we did here is, we believe, a useful approach to consider in further investigations. Behavioural change—particularly at the scale needed in the context of climate mitigation—typically occurs over time and through a series of stages (Tobler et al., 2011). The four action stages considered here (precontemplation, contemplation, preparation and action) paint a fuller and more nuanced picture than the oft-used dichotomous method (action or inaction), which in turn assists researchers in identifying the barriers that may be most pertinent at each phase and facilitates more targeted interventions to ‘nudge’ individuals through to the next stage.

## Conclusions

Engaging youth in adopting a low carbon lifestyle is an important component of efforts to limit global temperature rise to below 2 °C above pre-industrial levels. This study sought to better understand the current CC mitigation behaviour and intent amongst 17–18-year olds and the predictors for their action stages. Encouragingly, acceptance of CC and its anthropogenic origins are high, as is the belief that one’s individual activities/lifestyles can help to lessen it. While most youth report currently performing multiple mitigation actions, these are largely limited to lower-impact behaviours, such as recycling. Descriptive normative beliefs predict intent to adopt all of the high-impact actions assessed (*have no children or one less child, no car or first/next car will be electric, eat less meat*), and a more internal locus of control and lower CC scepticism are also predictive for some of these behaviours.

Education, policy and communications interventions around high-impact behaviours are encouraged, particularly those that aim to increase agency amongst youth. Concurrently, initiatives that make use of descriptive rather than injunctive norms may be very effective, including celebrity endorsement of target activities. Qualitative research is needed to better understand the attitudes and behavioural barriers around having no children or one less child and eating less meat. The Theory of Planned Behaviour and the Transtheoretical Model are useful frameworks for conceptualising and understanding individual-level GGE-reducing action, intent and barriers amongst youth and should allow for more targeted interventions in the future that seek to facilitate meaningful mitigation from this essential actor.

## Supplementary Information

Below is the link to the electronic supplementary material.

Supplementary file1 (DOCX 2.97 mb)

## References

[CR1] Ajzen I (1991). The theory of planned behavior. Organ Behav Hum Decis Process.

[CR2] Ajzen I (2002) Constructing a TpB questionnaire: Conceptual and methodological considerations. http://www.people.umass.edu/aizen/pdf/tpb.measurement.pdf. Accessed 3 Aug 2020

[CR3] Albrecht G (2011) Chronic environmental change: emerging ‘psychoterratic’ syndromes. In: Weissbecker I (ed) Climate Change and Human Well-being: Global Challenges and Opportunities. New York, pp 43–56. 10.1007/978-1-4419-9742-5_3

[CR4] Baiocchi G, Minx J, Hubacek K (2010). The impact of social factors and consumer behavior on carbon dioxide emissions in the United Kingdom. J Ind Ecol.

[CR5] Bandura A (1977). Self-efficacy: Toward a unifying theory of behavioral change. Psychol Rev.

[CR6] Barr S, Gilg AW (2007). A conceptual framework for understanding and analyzing attitudes towards environmental behaviour. Geogr Ann Ser B.

[CR7] Bland HW, Melton BF, Welle P, Bigham L (2012). Stress tolerance: new challenges for millennial college students. J Coll Stud Dev.

[CR8] Blasche J, Ketelaar PE (2015). The synergy in green persuasion: green celebrity endorsers in green advertising: a study of brand-endorser congruence effects in green advertising. Eur J Mark.

[CR9] Bulkeley H (2000). Common Knowledge? Public Understanding of Climate Change in Newcastle, Australia. Public Underst Sci.

[CR10] Caillaud S, Kalampalikis N (2013). Focus groups and ecological practices: a psychosocial approach. Qual Res Psychol.

[CR11] CDC (Centers for Disease control and Prevention) (1996) Guidelines for School Health Programs to Promote Lifelong Healthy Eating. MMWR*.* 45: 1–37 8637498

[CR12] Chan K, Yu LN, Luk EK (2013). Impact of celebrity endorsement in advertising on brand image among Chinese adolescents. Young Consum.

[CR13] Clayton S (2020). Climate anxiety: Psychological responses to climate change. J Anxiety Disord.

[CR14] Cliceri D, Spinelli S, Dinnella C, Prescott J, Monteleone E (2018). The influence of psychological traits, beliefs and taste responsiveness on implicit attitudes toward plant- and animal-based dishes among vegetarians, flexitarians and omnivores. Food Qual Prefer.

[CR15] Cooper PJ, Goodyer I (1997). Prevalence and significance of weight and shape concerns in girls aged 11–16 years. Br J Psychiatry.

[CR16] Cox DA (2019). The decline of religion in American family life. American Enterprise Institute. https://www.aei.org/research-products/report/the-decline-of-religion-in-american-family-life. Accessed 12 June 2020

[CR17] Dhont K, Hodson G (2014). Why do right-wing adherents engage in more animal exploitation and meat consumption?. Pers Individ Differ.

[CR18] Dietz T, Gardner GT, Gilligan J, Stern PC, Vandenbergh MP (2009). Household actions can provide a behavioral wedge to rapidly reduce US carbon emissions. PNAS.

[CR19] Elections Canada (2019) Official voting results forty-third general election. https://www.elections.ca/res/rep/off/ovr2019app/home.html. Accessed 8 April 2020

[CR20] Elgaaied-Gambiera L, Monnota E, Reniou F (2018). Using descriptive norm appeals effectively to promote green behavior. J Bus Res.

[CR21] Festinger L (1957) A Theory of Cognitive Dissonance (Vol. 2). California, Stanford university press

[CR22] Fielding KS, Head BW (2012). Determinants of young Australians’ environmental actions: The role of responsibility attributions, locus of control, knowledge and attitudes. Environ Educ Res.

[CR23] Fox N, Ward K (2008). Health, ethics and environment: A qualitative study of vegetarian motivations. Appetite.

[CR24] Gardner GT, Stern PC (2008). The Short List: The Most Effective Actions US Households Can Take to Curb Climate Change. Environ Sci Policy.

[CR25] Gehlbach H, Artino AR (2018). The survey checklist (manifesto) [Perspective]. Acad Med.

[CR26] Gidley JM, Bateman D, Smith C, Slaughter RA (2004). Futures in education: Principles, practice and potential Series 5.

[CR27] Gifford R (2011). The dragons of inaction: Psychological barriers that limit climate change mitigation and adaptation. Am Psychol.

[CR28] Girod B, Vuuren DPV, Hertwich EG (2014). Climate policy through changing consumption choices: Options and obstacles for reducing greenhouse gas emissions. Glob Environ Change.

[CR29] Government of Canada (2020) Electric vehicle and alternative fuel infrastructure deployment initiative. https://www.nrcan.gc.ca/energy-efficiency/energy-efficiency-transportation/electric-vehicle-alternative-fuels-infrastructure-deployment-initiative/18352. Accessed 3 June 2020

[CR30] Green J, Steinbach R, Garnett E, Christie N, Prior L (2018). Automobility reconfigured? Ironic seductions and mundane freedoms in 16–21 year olds’ accounts of car driving and ownership. Mobilities.

[CR31] Gromet DM, Kunreuther H, Larrick RP (2013). Political ideology affects energy-efficiency attitudes and choices. PNAS.

[CR32] Guth JL, Green JC, Kellstedt LA, Smidt CE (1995). Faith and the environment: religious beliefs and attitudes on environmental policy. Am J Polit Sci.

[CR33] Halvorson B (2020) Cost remains the biggest barrier against EV adoption, study finds. https://www.greencarreports.com/news/1126706_cost-remains-the-biggest-barrier-against-ev-adoption-study-finds. Accessed 3 June 2020

[CR35] Hine DW, Gifford R (1996). Individual restraint and group efficiency in commons dilemmas: the effects of two types of environmental uncertainty. J Appl Soc Psychol.

[CR36] Inoue Y, Kent A (2012). Sport teams as promoters of pro-environmental behavior: an empirical study. J Sport Manag.

[CR37] IPCC (2018) Global Warming of 1.5°C. An IPCC Special Report on the impacts of global warming of 1.5°C above pre-industrial levels and related global greenhouse gas emission pathways, in the context of strengthening the global response to the threat of climate change, sustainable development, and efforts to eradicate poverty (Rep) Masson- Delmotte V, Zhai P, Pörtner H, Roberts D, Skea J, Shukla P, et al. (eds)

[CR38] Kamins MA, Gupta K (1994). Congruence between spokesperson and product type: A matchup hypothesis perspective. Psychol Mark.

[CR39] Khalid NL, Jayasainan SY, Hassim N (2018). Social media influencers - shaping consumption culture among Malaysian youth. International Conference on Humanities and Social Sciences.

[CR40] Lacroix K (2018). Comparing the relative mitigation potential of individual pro-environmental behaviors. J Clean Prod.

[CR41] Leeuw AD, Valois P, Ajzen I, Schmidt P (2015). Using the theory of planned behavior to identify key beliefs underlying pro-environmental behavior in high-school students: Implications for educational interventions. J Environ Psychol.

[CR42] Leiserowitz A, Maibach EW, Roser-Renouf C, Feinberg G, Rosenthal S (2014). Climate Change in the American Mind: Americans' Global Warming Beliefs and Attitudes in April 2013. SSRN.

[CR43] Lipka M (2018) 5 facts about religion in Canada. https://www.pewresearch.org/fact-tank/2019/07/01/5-facts-about-religion-in-canada. Accessed 8 April 2020

[CR44] Liu X, Stoutenborough J, Vedlitz A (2017). Bureaucratic expertise, overconfidence, and policy choice. Governance.

[CR45] Maki A, Burns RJ, Ha L, Rothman AJ (2016). Paying people to protect the environment: A meta-analysis of financial incentive interventions to promote proenvironmental behaviors. J Environ Psychol.

[CR46] Martin CA, Bush AJ (2000). Do role models influence teenagers’ purchase intentions and behavior?. J Consum Mark.

[CR47] Mcneill KL, Vaughn MH (2010). Urban High School Students’ Critical Science Agency: Conceptual Understandings and Environmental Actions Around Climate Change. J Res Sci Teach.

[CR48] Melnyk V, Van Herpen E, Van Trijp H (2010) The Influence of Social Norms in Consumer Decision Making: A Meta-Analysis. In: Campbell MC, Inman J, Pieters R (Eds) Advances in Consumer Research, Vol 37. Duluth, MN, pp 463–464

[CR49] Moran D, Wood R, Hertwich E, Mattson K, Rodriguez JFD, Schanes K, Barrett J (2020). Quantifying the potential for consumer-oriented policy to reduce European and foreign carbon emissions. Clim Policy.

[CR50] Morrison M, Duncan R, Parton K (2015) Religion Does Matter for Climate Change Attitudes and Behavior. PloS One 10(8). 10.1371/journal.pone.0134868 10.1371/journal.pone.0134868 PMC4527763 26247206

[CR51] Mostafa MM (2016). Post-materialism, Religiosity, Political Orientation, Locus of Control and Concern for Global Warming: A Multilevel Analysis Across 40 Nations. Soc Indic Res.

[CR52] Munoz-Carrier G, Thomsen D, Pickering GJ (2020). Psychological and experiential factors affecting climate change perception: learnings from a transnational empirical study and implications for framing climate-related flood events. Environ Res Commun.

[CR53] Murtaugh PA, Schlax MG (2009). Reproduction and the carbon legacies of individuals. Glob Environ Change.

[CR54] Nielsen KS, Cologna V, Lange F, Brick C, Stern PC (2021) The case for impact focused environmental psychology. J Environ Psychol, 101559. 10.1016/j.jenvp.2021.101559

[CR55] Pacala S, Socolow R (2004). Stabilization wedges: solving the climate problem for the next 50 years with current technologies. Science.

[CR56] Palupi T, Sawitri DR (2018). The importance of pro-environmental behavior in adolescent. Web Conf.

[CR57] Parizeau K, von Massow M, Martin R (2015). Household-level dynamics of food waste production and related beliefs, attitudes, and behaviours in Guelph, Ontario. Waste Manage.

[CR58] Pearce LD, Hayward GM, Pearlman JA (2017). Measuring five dimensions of religiosity across adolescence. Rev Relig Res.

[CR59] Pedersen RL, Lam DP (2018). Second comment on ‘The climate mitigation gap: Education and government recommendations miss the most effective individual actions’. Environ Res Lett.

[CR60] Peters E, Västfjäll D, Slovic P, Mertz C, Mazzocco K, Dickert S (2006). Numeracy and Decision Making. Psychol Sci.

[CR61] Pew Research Center (2018) Young adults around the world are less religious by several measures. https://www.pewforum.org/2018/06/13/young-adults-around-the-world-are-less-religious-by-several-measures. Accessed 12 June 2020

[CR62] Pickering G (2015). Head in the (oil) sand? climate change scepticism in Canada. Enviro Soc Sci.

[CR63] Pickering GJ, Pickering HM, Northcotte A, Habermebl C (2020). Participation in residential organic waste diversion programs: Motivators and optimizing educational messaging. Resour Conserv Recycl.

[CR64] Pickering GJ, Schoen K, Botta M, Fazio Z (2020). Exploration of youth knowledge and perceptions of individual-level climate mitigation action. Environ Res Lett.

[CR65] Poortinga W, Spence A, Whitmarsh L, Capstick S, Pidgeon NF (2011). Uncertain climate: An investigation into public scepticism about anthropogenic climate change. Glob Environ Change.

[CR66] Prochaska JO, Velicer WF (1997). The transtheoretical model of health behavior change. Am J Health Promot.

[CR67] Prochaska JO, Redding CA, Evers KE (2008) The transtheoretical model and stages of change. In: Glanz K, Rimer BK, Viswanath K (Eds) Health behavior and health education: Theory, research, and practice 4th edn. San Francisco, CA, pp 97–121

[CR68] Rutjens B, Sutton R, Van der Lee R (2018). Not all skepticism is equal: exploring the ideological antecedents of science acceptance and rejection. Pers Soc Psychol Bull.

[CR69] Sarabia L (2020) Canada's EV charging networks are growing at pace, but more is needed. https://electricautonomy.ca/2020/03/02/canadas-ev-charging-networks-2020. Accessed 3 June 2020

[CR70] Schanes K, Dobernig K, Gözet B (2018). Food waste matters - A systematic review of household food waste practices and their policy implications. J Clean Prod.

[CR71] Shi J, Visschers VHM, Siegrist M, Arvai J (2016). Knowledge as a driver of public perceptions about climate change reassessed. Nature Clim Change.

[CR72] Smith N, Leiserowitz A (2013). American evangelicals and global warming. Glob Environ Change.

[CR73] Sparrow J (2019) The car culture that’s helping destroy the planet was by no means inevitable. https://lithub.com/the-car-culture-thats-helping-destroy-the-planet-was-by-no-means-inevitable. Accessed 12 June 2020

[CR74] Stafford R, Jones PJ (2019). Viewpoint – Ocean plastic pollution: A convenient but distracting truth?. Mar Policy.

[CR75] Statista (2016) Meat consumption in Canada from 2013–2015. https://www.statista.com/statistics/521135/meat-consumption-canada. Accessed 12 June 2020

[CR76] Stea S, Pickering GJ (2018). Optimizing messaging to reduce red meat consumption. Environ Commun.

[CR77] Stern PC, Wolske KS (2017). Limiting climate change: What’s most worth doing?. Environ Res Lett.

[CR78] Stoutenborough JW, Vedlitz A (2014). The effect of perceived and assessed knowledge of climate change on public policy concerns: An empirical comparison. Enivron Sci Policy.

[CR79] Tjernström E, Tietenberg T (2008). Do differences in attitudes explain differences in national climate change policies?. Ecol Econ.

[CR80] Tobler C, Visschers VH, Siegrist M (2011). Eating green. Consumers’ willingness to adopt ecological food consumption behaviors. Appetite.

[CR81] Tonglet M, Phillips PS, Bates MP (2004). Determining the drivers for householder pro-environmental behaviour: waste minimisation compared to recycling. Resour Conserv Recycl.

[CR82] Tonn B, Macgregor D (2009). Individual approaches to futures thinking and decision making. Futures.

[CR83] United Nations (2020). Emissions Gap Report 2020. United Nations Environment Programme. Nairobi. https://www.unep.org/emissions-gap-report-2020. Accessed 29 June 2021

[CR84] Van De Ven D-J, González-Eguino M, Arto I (2018). The potential of behavioural change for climate change mitigation: a case study for the European Union. Mitig Adapt Strateg Glob Chang.

[CR85] Wardle J, Haase AM, Steptoe A, Nillapun M, Jonwutiwes K, Bellisie F (2004). Gender differences in food choice: The contribution of health beliefs and dieting. Ann Behav Med.

[CR86] Whitmarsh L, Seyfang G, O’Neill S (2011). Public engagement with carbon and climate change: To what extent is the public ‘carbon capable’?. Glob Environ Change.

[CR87] Wynes S, Nicholas KA (2017) The climate mitigation gap: Education and government recommendations miss the most effective individual actions. Environ Res Lett (12). 10.1088/1748-9326/aa7541

[CR88] Wynes S, Nicholas KA (2018). Reply to Comment on ‘The climate mitigation gap: Education and government recommendations miss the most effective individual actions’. Environ Res Lett.

[CR89] Wynes S, Nicholas KA (2019) Climate science curricula in Canadian secondary schools focus on human warming, not scientific consensus, impacts or solutions. PloS One 14(7). 10.1371/journal.pone.0218305 10.1371/journal.pone.0218305 PMC6639000 31318862

[CR90] Wynes S, Zhao J, Donner SD (2020). How well do people understand the climate impact of individual actions?. Climatic Change.

