# Supplementary material for: Lifestyle decisions and climate mitigation: current action and behavioural intent of youth
Source: Mitig Adapt Strateg Glob Chang. 2021 Jul 26;26(6):25. doi: 10.1007/s11027-021-09963-4 (PMC8550525; doi:10.1007/s11027-021-09963-4)
Supplement: Supplementary file 1 — Supplementary file1 (DOCX 2.97 mb) [file 11027_2021_9963_MOESM1_ESM.docx]

Journal name: Mitigation and Adaptation Strategies for Global Change

Article title: Lifestyle decisions and climate mitigation: current action and behavioural intent of youth

Author names: Gary J. Pickering, Kaylee Schoen, Marta Botta

Corresponding author: Gary J. Pickering (gpickering@brocku.ca)

**Online Appendices**

**Climate change uncertainty and skepticism (statements from Poortinga et al. 2011)**

*I am unsure that climate change is really happening*

*Most scientists agree that humans are causing climate change**

*The seriousness of climate change is exaggerated*

*It is uncertain what the effects of climate change will be.*

^*^ reverse coded for analysis purposes

**Response options for recognition of the anthropogenic origins of climate change**

*Climate change is entirely caused by natural processes*

*Climate change is mainly caused by natural processes*

*Climate change is partly caused by natural processes and partly caused by human activity*

*Climate change is mainly caused by human activity*

*Climate change is completely caused by human activity.*

**Determining religious exclusivity (Pearce et al. 2017)**

*Q1 Is it okay for religious people to try to convert other people to their faith, or should everyone leave everyone else alone?*

*Q2 Do you think it is okay for someone of your religion to also practice other religions, or should people only practice one religion?*

*Q3 Which of the following statements comes closest to your own views about religion?:*

*- Truth is not in one religion*

*- Only one religion is true*

*Q4 Some people think that it is okay to pick and choose their religious beliefs without having to accept the teachings of their religious faith as a whole. Do you agree or disagree?*

**Determining religious salience (Pearce et al. 2017)**

*Q1 If you were unsure of what was right or wrong in a particular situation, how would you decide what to do?*

*- Something other than God or scripture*

*- Do what God or scripture says is right*

*Q2 Have you ever made a personal commitment to live your life for God?*

*- Did not make commitment to live for God*

*- Made commitment to live for God*

*Q3 How important or unimportant is religious faith in shaping how you live your daily life?*

*- Not important at all*

*- Not very important*

*- Somewhat important*

*- Very important*

*- Extremely important*

Table 1. **Distribution of scores for (A) objective climate change knowledge (% of questions answered correctly), (B) climate change scepticism (higher values correspond to greater scepticism), and (C) locus of control (LOC) for environmental action (higher scores indicate greater internal LOC, lower scores indicate greater external LOC) (N = 476)**


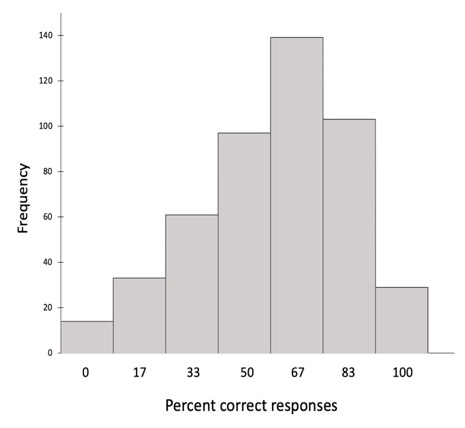

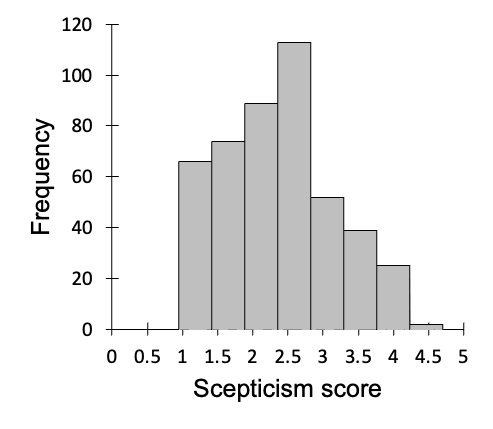


Frequency

Frequency

Frequency

Objective climate change knowledge

Climate change scepticism score

Locus of control score

A

B

C

**Calculations and sources supporting Figure 2 (emissions savings per individual per year (tCO_2_) for specific actions)**

***Recycle***: 0.21. Soured from Murtaugh and Schlax (2009).

***Take Public Transportation***: No value given. The savings in GGE are too uncertain due to high variability in the specific circumstances of and options available to each individual (e.g. is the alternative to take a fossil-fuel powered car, an electric car, cycle?) and the energy source used by the public transport.

***Conserve energy in the home***: 0.63. Wynes and Nicholas (2017; Supplementary Data) calculate that the energy used by the average Canadian household produces 6.286 tCO_2_e/year. Assuming that an overall reduction of 25% in energy use is achievable, per the US Department of Energy (ND), this equates to a saving of 1.57 tCO_2_e/year. The average Canadian household size is 2.5, so an individual can save approximately 0.63 tCO_2_e/year (1.57/2.5) through energy conservation efforts at home.

***Conserve water****:* 0.15. Assuming average domestic household water use contributes 942kg CO_2_e/yr (Ro, 2020). Up to approximately 40% saving in water use could be realistically achieved using the examples and figures cited in Ro (2020). 40% of 942 = 377, which divided by the average Canadian household size (2.5) gives 151kg (0.15t) CO_2_e/year.

***No children or one less child***: 58.6. Sourced from Wynes and Nicholas (2017) and based on the average of three studies and countries. We note that of these studies, the value for the USA – the country closest to Canada geographically and culturally – is 118. Thus, 58.6 tCO_2_e/year may underestimate the ‘true’ savings in the Canadian context.

***Vacation locally instead of flying to destination***: 0.6. Based on a return flight from Toronto to Orlando (1697 km, economy class) per the average of 11 studies reviewed in the Supplementary Data of Wynes and Nicholas (2017).

***Avoid Products with Excessive Packaging***: No value given. No suitable studies or data were identified that could be used to reasonably approximate a value for this action.

***No car or first/next car will be electric***: 1.68. Average value of living car-free in Canada (2.2; Zamel and Li 2006) and switching to an electric car (1.15; five country average from Wynes and Nicholas 2017).

***Eat less meat***: 0.8. Based on the average value across five studies from Wynes and Nicholas (2017) for the ‘Adopt a plant-based diet’ action. We recognise that this is only an approximation and perhaps overestimates actual savings given that the two behaviours are not identical and that *Eat less meat* was not quantified in our study.

Table 2. Correlation (Pearson) matrix for measures used in the survey (n = 285-482). Values in bold are different from zero (p(r)<0.05).

| Variables | Subjective Knowledge | Objective Knowledge | Efficacy Knowledge | Locus of Control | CC Scepticism | Religious Exclusivity | Religious Salience |
| --- | --- | --- | --- | --- | --- | --- | --- |
| Subjective CC Knowledge | **1** | **0.227** | -0.010 | **0.144** | -0.087 | 0.025 | **0.162** |
| Objective CC Knowledge | **0.227** | **1** | **0.100** | **0.198** | **-0.233** | -0.073 | -0.019 |
| Knowledge of Action Efficacy | -0.010 | **0.100** | **1** | 0.065 | **-0.141** | -0.029 | -0.040 |
| Locus of Control | **0.144** | **0.198** | 0.065 | **1** | **-0.382** | -0.081 | 0.038 |
| CC Scepticism | -0.087 | **-0.233** | **-0.141** | **-0.382** | **1** | **0.162** | **0.170** |
| Religious Exclusivity | 0.025 | -0.073 | -0.029 | -0.081 | **0.162** | **1** | **0.591** |
| Religious Salience | **0.162** | -0.019 | -0.040 | 0.038 | **0.170** | **0.591** | **1** |

**Figure 1. Distribution of number of climate mitigation behaviours (Figure 1) for which youth are currently in the *Action* stage (N = 463)**

**Table 3. Summary statistics for descriptive normative beliefs for high impact climate mitigation actions**

Eat Less Meat

No Car or First/Next Car will be Electric

No Children or Have One Fewer Child

**Figure 2. Distribution of descriptive normative belief (‘social norm’) scores**

(scores are averaged across referent others)

No children or one less child

.

No car or first/next car will be electric Eat less meat
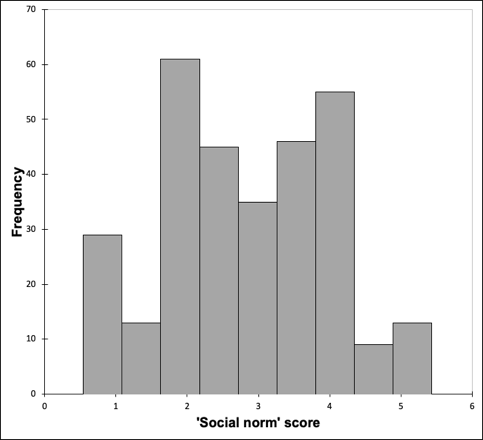


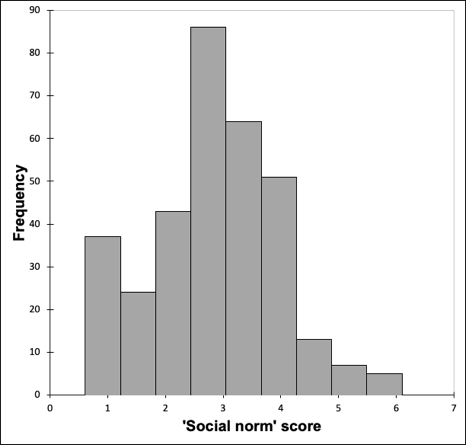


**Figure 3. Distribution of Efficacy Knowledge Scores (EKS)** (n = 448)


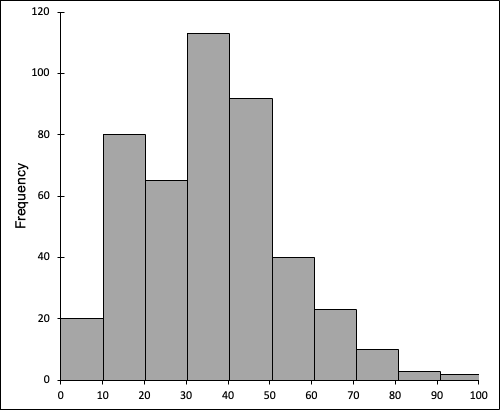


Table 4. Multinomial regression for willingness to have one fewer child or not intending to have children, with standardised β-values (β), standard errors (SEs), odds ratios (ORs), and 95% conﬁdence intervals (95% CIs).

|  | Precontemplation (n = 169) | | | Action (n = 157) | | |
| --- | --- | --- | --- | --- | --- | --- |
|  | β (SE) | OR | 95% CI | β (SE) | OR | 95% CI |
| Gender (male) | -.03 (.07) | .89 | -.17-.11 | -.07 (.07) | .79 | -.20-.07 |
| Age | **.15**^*^ (.07) | 1.7 | .00-.29 | -.02 (.07) | .92 | -.16-.12 |
| Religious affiliation (yes) | .02 (.07) | 1.1 | -.13-.16 | -.06 (.07) | .81 | -.20-.09 |
| Subjective climate change knowledge | -.00 (.08) | .99 | -.16-.15 | -.11 (.08) | .80 | -.26-.04 |
| Objective climate change knowledge | -.01 (.08) | 1.0 | -.16-.14 | -.08 (.08) | .99 | -.23-.07 |
| Knowledge of mitigation efficacy (EKS) | -.13 (.08) | .99 | -.23-.02 | .04 (.07) | 1.00 | -.10-.18 |
| Social norm | **-.41**^***^ (.08) | .54 | -.56- -.25 | .11 (.08) | 1.18 | -.04-.26 |
| Locus of control | -.03 (.08) | .93 | -.19-.13 | -.06 (.08) | .88 | -.22-.10 |
| Climate change scepticism | .07 (.08) | 1.2 | -.09-.23 | -.01 (.08) | .98 | -.16-.15 |

Reference category for the multinomial regression was the change (contemplation/preparation) stages (n = 108); significant coefficients (βs) are shown in bold; * p < .05, ** p < .01, *** p < .001

Table 5. Multinomial regression for “my first/next car will be electric” or “I do not intend to purchase a car”, with standardised β-values (β), standard errors (SEs), odds ratios (ORs), and 95% conﬁdence intervals (95% CIs).

|  | Precontemplation (n = 63) | | | Action (n = 142) | | |
| --- | --- | --- | --- | --- | --- | --- |
|  | β (SE) | OR | 95% CI | β (SE) | OR | 95% CI |
| Gender (male) | .05 (.08) | 1.19 | -.12-.21 | -.11 (.06) | 0.66 | -.23-.01 |
| Age | .09 (.09) | 1.40 | -.08-.26 | -.02 (.06) | 0.93 | -.14-.10 |
| Religious affiliation (yes) | .07 (.09) | 1.32 | -.09-.24 | .02 (.06) | 1.08 | -.10-.14 |
| Subjective climate change knowledge | -.15 (.08) | 0.74 | -.32-.01 | .06 (.07) | 1.13 | -.07-.19 |
| Objective climate change knowledge | .07 (.09) | 1.01 | -.10-.24 | .05 (.07) | 1.00 | -.08-.18 |
| Knowledge of mitigation efficacy (EKS) | .06 (.09) | 1.01 | -.12-.23 | .**20**^***^ (.06) | 1.02 | .08-.33 |
| Social norm | **-.39**^***^ (.09) | 0.53 | -.57- -.21 | **-.13**^*^ (.07) | 0.80 | -.26-.00 |
| Locus of control | -.06 (.09) | 0.87 | -.24-.11 | .00 (.07) | 0.99 | -.14-.13 |
| Climate change scepticism | **.38**^***^ (.10) | 2.33 | .18-.58 | .06 (.07) | 1.14 | -.08-.20 |

Reference category for the multinomial regression was the change (contemplation/preparation) stages (n = 232); significant coefficients (βs) are shown in bold; * p < .05, ** p < .01, *** p < .001

Table 6. Multinomial regression for willingness to eat less meat, with standardised β-values (β), standard errors (SEs), odds ratios (ORs), and 95% conﬁdence intervals (95% CIs).

|  | Precontemplation (n = 166) | | | Action (n = 118) | | |
| --- | --- | --- | --- | --- | --- | --- |
|  | β (SE) | OR | 95% CI | β (SE) | OR | 95% CI |
| Gender (male) | .11 (.07) | 1.49 | -.02-.24 | **-.15**^*^ (.07) | 0.57 | -.30- -.01 |
| Age | **.26**^***^ (.07) | 2.64 | .13-.39 | .11 (.07) | 1.50 | -.03-.25 |
| Religious affiliation (yes) | -.07 (.07) | 0.77 | -.20-.06 | -.04 (.07) | 0.85 | -.19-.10 |
| Subjective climate change knowledge | .08 (.07) | 1.18 | -.05-.22 | **.15**^*^ (.08) | 1.36 | .00-.31 |
| Objective climate change knowledge | .02 (.07) | 1.00 | -.11-.16 | -.07 (.08) | 0.99 | -.22-.09 |
| Knowledge of mitigation efficacy (EKS) | .07 (.07) | 1.01 | -.06-.21 | **.21^**^** (.07) | 1.02 | .06-.35 |
| Social norm | **-.25**^***^ (.07) | 0.64 | -.39- -.11 | .11 (.08) | 1.22 | -.04-.26 |
| Locus of control | -.09 (.07) | 0.81 | -.24-.05 | **.18^*^** (.08) | 1.49 | .01-.34 |
| Climate change scepticism | .09 (.07) | 1.24 | -.05-.24 | -.04 (.08) | 0.92 | -.19-.12 |

Reference category for the multinomial regression was the change (contemplation/preparation) stages (n = 149); significant coefficients (βs) are shown in bold; * p < .05, ** p < .01, *** p < 0.001

Table 7. Multinomial regression for willingness to recycle, with standardised β-values (β), standard errors (SEs), odds ratios (ORs), and 95% conﬁdence intervals (95% CIs).

|  | Precontemplation (n = 13) | | | Action (n = 366) | | |
| --- | --- | --- | --- | --- | --- | --- |
|  | β (SE) | OR | 95% CI | β (SE) | OR | 95% CI |
| Gender (male) | -.04 (.17) | 0.86 | -.37-.29 | -.10 (.07) | 0.70 | -.24-.04 |
| Age | .22 (.22) | 2.30 | -.22 -.66 | **-.19**^*^ (.08) | 0.49 | -.34- -.04 |
| Religious affiliation (yes) | .05 (.19) | 1.23 | -.32-.43 | -.10 (.08) | 0.69 | -.25-.05 |
| Subjective climate change knowledge | .01 (.16) | 1.02 | -.31-.33 | .12 (.07) | 1.28 | -.02-.27 |
| Objective climate change knowledge | -.18 (.17) | 0.99 | -.52-.15 | .05 (.07) | 1.00 | -.09-.20 |
| Knowledge of mitigation efficacy (EKS) | .31 (.20) | 1.03 | -.08-.70 | .05 (.08) | 1.01 | -.10-.20 |
| Locus of control | -.06 (.18) | 0.86 | -.43-.30 | .12 (.08) | 1.32 | -.03-.27 |
| Climate change scepticism | .28 (.22) | 1.86 | -.15-.70 | **-.30** ^***^ (.08) | 0.51 | -.47- -.14 |

Reference category for the multinomial regression was the change (contemplation/preparation) stages (n = 79); significant coefficients (βs) are shown in bold; * p < .05, ** p < .01, *** p < .001

Table 8. Multinomial regression for willingness to take public transport, with standardised β-values (β), standard errors (SEs), odds ratios (ORs), and 95% conﬁdence intervals (95% CIs).

|  | Precontemplation (n = 68) | | | Action (n = 249) | | |
| --- | --- | --- | --- | --- | --- | --- |
|  | β (SE) | OR | 95% CI | β (SE) | OR | 95% CI |
| Gender (male) | -.07 (.09) | 0.79 | -.24.10 | -.04 (.06) | 0.86 | -.17-.08 |
| Age | .09 (.09) | 1.41 | -.08-.27 | .00 (.06) | 0.99 | -.13-.12 |
| Religious affiliation (yes) | .10 (.09) | 1.47 | -.07-.28 | .01 (.06) | 1.05 | -.11-.14 |
| Subjective climate change knowledge | .04 (.09) | 1.08 | -.14-.22 | .01 (.06) | 1.02 | -.12-.13 |
| Objective climate change knowledge | -.12 (.09) | 0.99 | -.29-.05 | .02 (.07) | 1.00 | -.12-.15 |
| Knowledge of mitigation efficacy (EKS) | -.07 (.09) | 0.99 | -.26-.11 | .05 (.06) | 1.01 | -.07-.18 |
| Locus of control | **-.22**^*^ (.09) | 0.61 | -.40- -.03 | -.06 (.07) | 0.87 | -.20-.08 |
| Climate change scepticism | -.03 (.09) | 0.94 | -.21-.16 | **-.15**^*^ (.07) | 0.71 | -.29- -.01 |

Reference category for the multinomial regression was the change (contemplation/preparation) stages (n = 119); significant coefficients (βs) are shown in bold; * p < .05, ** p < .01, *** p < .001

Table 9. Multinomial regression for willingness to conserve energy in the home, with standardised β-values (β), standard errors (SEs), odds ratios (ORs), and 95% conﬁdence intervals (95% CIs).

|  | Precontemplation (n = 16) | | | Action (n = 247) | | |
| --- | --- | --- | --- | --- | --- | --- |
|  | β (SE) | OR | 95% CI | β (SE) | OR | 95% CI |
| Gender (male) | .26 (.15) | 2.60 | -.04-.56 | .10 (.06) | 1.45 | -.01-21 |
| Age | .28 (.19) | 2.87 | -.08-.65 | **-.12**^*^ (.06) | 0.65 | -.23- -.01 |
| Religious affiliation (yes) | .08 (.17) | 1.35 | -.25-.40 | .09 (.06) | 1.41 | -.02-.20 |
| Subjective climate change knowledge | .08 (.16) | 1.17 | -.23-.39 | .05 (.06) | 1.11 | -.06-.17 |
| Objective climate change knowledge | -.10 (.15) | 0.99 | -.39-.19 | .09 (.06) | 1.01 | -.02-.21 |
| Knowledge of mitigation efficacy (EKS) | .02 (.18) | 1.00 | -.34-.37 | -.04 (.06) | 1.00 | -.15-.07 |
| Locus of control | -.18 (.17) | 0.66 | -.51-.14 | .08 (.06) | 1.21 | -.03-.20 |
| Climate change scepticism | **.46**^*^ (.19) | 2.80 | .09-.84 | **-.13**^*^ (.06) | 0.74 | -.26- -.01 |

Reference category for the multinomial regression was the change (contemplation/preparation) stages (n = 195); significant coefficients (βs) are shown in bold; * p < .05, ** p < .01, *** p < .001

Table 10. Multinomial regression for willingness to conserve water with standardised β-values (β), standard errors (SEs), odds ratios (ORs), and 95% conﬁdence intervals (95% CIs).

|  | Precontemplation (n = 32) | | | Action (n = 222) | | |
| --- | --- | --- | --- | --- | --- | --- |
|  | β (SE) | OR | 95% CI | β (SE) | OR | 95% CI |
| Gender (male) | .11 (.11) | 1.51 | -.10-.33 | .08 (.06) | 1.35 | -.03-.19 |
| Age | -.03 (.11) | 0.90 | -.25-.20 | **-.19**^***^ (.06) | 0.50 | -.30- -.08 |
| Religious affiliation (yes) | .04 (.12) | 1.16 | -.19-.27 | .00 (.06) | 0.99 | -.11-.11 |
| Subjective climate change knowledge | -.14 (.11) | 0.76 | -.35-.07 | .09 (.06) | 1.20 | -.02-.20 |
| Objective climate change knowledge | -.21 (.11) | 0.98 | -.43-.01 | -.01 (.06) | 1.00 | -.13-.11 |
| Knowledge of mitigation efficacy (EKS) | -.10 (.12) | 0.99 | -.34-.14 | .00 (.06) | 1.00 | -.11-.11 |
| Locus of control | -.10 (.12) | 0.79 | -.33-.12 | .08 (.06) | 1.20 | -.04-.20 |
| Climate change scepticism | .24 (.13) | 1.69 | -.02-.49 | -.03 (.06) | 0.93 | -.15-.09 |

Reference category for the multinomial regression was the change (contemplation/preparation) stages (n = 204); significant coefficients (βs) are shown in bold; * p < .05, ** p < .01, *** p < .001

Table 11. Multinomial regression for willingness to vacation locally with standardised β-values (β), standard errors (SEs), odds ratios (ORs), and 95% conﬁdence intervals (95% CIs).

|  | Precontemplation (n = 136) | | | Action (n = 150) | | |
| --- | --- | --- | --- | --- | --- | --- |
|  | β (SE) | OR | 95% CI | β (SE) | OR | 95% CI |
| Gender (male) | -.05 (.07) | 0.85 | -.18-.09 | -.06 (.07) | 0.79 | -.19-.06 |
| Age | **.17**^*^ (.07) | 1.87 | .03-.30 | -.01 (.07) | 0.97 | -.14-.12 |
| Religious affiliation (yes) | -.07 (.07) | 0.78 | -.20-.07 | **-.15**^*^ (.07) | 0.57 | -.28- -.02 |
| Subjective climate change knowledge | -.05 (.07) | 0.91 | -.18-.09 | .02 (.07) | 1.05 | -.11-.16 |
| Objective climate change knowledge | .04 (.07) | 1.00 | -.10-.18 | .03 (.07) | 1.00 | -.11-.17 |
| Knowledge of mitigation efficacy (EKS) | .06 (.07) | 1.01 | -.08-.19 | .10 (.07) | 1.01 | -.04-.23 |
| Locus of control | -.11 (.07) | 0.78 | -.26-.03 | .05 (.07) | 1.11 | -.10-.19 |
| Climate change scepticism | -.01 (.07) | 0.98 | -.16-.14 | -.02 (.07) | 0.96 | -.16-.12 |

Reference category for the multinomial regression was the change (contemplation/preparation) stages (n = 149); significant coefficients (βs) are shown in bold; * p < .05, ** p < .01, *** p < .001

Table 12. Multinomial regression for willingness to avoid products with excessive packaging, with standardised β-values (β), standard errors (SEs), odds ratios (ORs), and 95% conﬁdence intervals (95% CIs).

|  | Precontemplation (n = 49) | | | Action (n = 154) | | |
| --- | --- | --- | --- | --- | --- | --- |
|  | β (SE) | OR | 95% CI | β (SE) | OR | 95% CI |
| Gender (male) | -.01 (.09) | 0.95 | -.19-.16 | .01 (.06) | 1.03 | -.11-.12 |
| Age | .06 (.09) | 1.24 | -.12-.24 | **-.13**^*^ (.06) | 0.61 | -.25- -.02 |
| Religious affiliation (yes) | .09 (.10) | 1.42 | -.09-.28 | .01 (.06) | 1.02 | -.11-.12 |
| Subjective climate change knowledge | -.01 (.09) | 0.98 | -.19-.17 | .07 (.06) | 1.16 | -.05-.20 |
| Objective climate change knowledge | .01 (.09) | 1.00 | -.17-.18 | **.16**^*^ (.06) | 1.01 | .03-.29 |
| Knowledge of mitigation efficacy (EKS) | -.14 (.10) | 0.99 | -.33-.05 | -.07 (.06) | 0.99 | -.19-.05 |
| Locus of control | **-.23**^*^ (.10) | 0.59 | -.42- -.04 | **.14**^*^ (.07) | 1.37 | .01-.27 |
| Climate change scepticism | .12 (.10) | 1.31 | -.07-.31 | -.01 (.06) | 0.97 | -.14-.11 |

Reference category for the multinomial regression was the change (contemplation/preparation) stages (n = 256); significant coefficients (βs) are shown in bold; * p < .05, ** p < .01, *** p < .001
